# The Posture Detection Method of Caged Chickens Based on Computer Vision

**DOI:** 10.3390/ani14213059

**Published:** 2024-10-24

**Authors:** Cheng Fang, Xiaolin Zhuang, Haikun Zheng, Jikang Yang, Tiemin Zhang

**Affiliations:** 1 College of Engineering, South China Agricultural University, 483 Wushan Road, Guangzhou 510642, China; gu5457111@gmail.com (C.F.);; 2 School of Mechanical and Energy Engineering, Guangdong Ocean University, Yangjiang 529500, China; 3 National Engineering Research Center for Breeding Swine Industry, Guangzhou 510642, China; 4 Guangdong Laboratory for Lingnan Modern Agriculture, Guangzhou 510642, China

**Keywords:** caged chicken, computer vision, depth image, posture detection, smart agriculture

## Abstract

At present, raising caged chickens is a common farming method in China. However, monitoring the status of caged chickens is still done by human labor, which is time-consuming and laborious. This paper proposed a posture detection method for caged chickens based on computer vision, which can automatically identify the standing and lying posture of chickens in a cage. For this aim, an image correction method was used to rotate the image and make the feeding trough horizontal in the image. The variance method and the speeded-up robust features method were proposed to identify the feeding trough and indirectly obtain the key area through the feeding trough position. In this paper, a depth camera was used to generate three-dimensional information so that it could extract the chickens from the image of the key area. After some constraint conditions, the chickens’ postures were screened. The experimental results show that the algorithm can achieve 97.80% precision and 80.18% recall (IoU > 0.5) for white chickens and can achieve 79.52% precision and 81.07% recall (IoU > 0.5) for jute chickens (yellow and black feathers). It runs at ten frames per second on an i5-8500 CPU. Overall, the results indicated that this study provides a non-invasive method for the analysis of posture in caged chickens, which may be helpful for future research on poultry.

## Simple Summary

This system can remind staff to do a further inspection of broilers, which are kept in a lying posture for a long time. This system can provide early warning of the health status of caged broilers and provide a reference for subsequent poultry automated disease detection. In addition, this method will help staff monitor their animals’ behavior and health more efficiently.

## 1. Introduction

Caged chickens, compared to non-caged chickens, have a much higher production efficiency. It makes full use of building space and reduces farming costs, thus effectively relieving the pressure of land resource shortage [1,2,3]. For large caged chicken farms, due to a large number of chickens and relatively few staff, the inspection of the chickens’ health status is rather difficult. Meanwhile, chickens get sick or die every day on the farm, with a death and culling rate 5% higher than for non-caged chickens. Thus, the late discovery of sick chickens could result in a large scale infection among the chickens, leading to serious economic loss and even threatening human health. For this reason, the early warning of poultry disease has drawn serious attention from poultry farmers [4,5].

In recent years, the welfare of animals has received increasing research attention. Some researchers used automated monitoring equipment to study the behavioral and physiological response of laying hens [6]. Other researchers used thermal images in a broiler flock to develop a temperature measurement system [7]. Still other researchers used sound technology to automatically detect the feeding behaviors of broilers [8] or to predict the growth of broilers [9]. The research could be used as an early warning method or a continuous monitoring system to evaluate the health status of the chickens.

With the rise of computer vision, more poultry studies have emerged [10]. Some image algorithms were used to identify the poultry [11]. Some were used to estimate their live weight [12]. More algorithms are concerned with their behavior [13]. Some algorithms could classify the poultry behavior, such as running, standing, walking, eating, resting, and preening [14,15,16]. Cameras could also monitor the movement of broilers [17,18,19], but this is limited to non-caged chickens, and rearing caged chickens becomes more complicated.

As an indicator of health state, chicken posture has always been the focus and difficulty of research. Due to the high density of caged chickens and lack of light on the farm, it is difficult to accurately identify the chickens by computer vision. The camera could be used to identify the chicken’s feet in the cage and indirectly tell whether the chicken was standing or lying down. However, in this experiment, the result was not ideal due not only to the dim light on the farm but also to the blocking of the feeding trough, making it even dimmer inside the cage. Adding a supplementary light source to the farm is also not feasible, since that will change the environment where the chickens grow and may affect their growth.

Three-dimensional (3D) vision cameras could be used to estimate the weight of poultry or livestock [20,21], and these kinds of cameras could also recognize their behaviors automatically by image algorithms [22,23]. In this paper, a depth camera was used to simultaneously acquire both colored and depth images, and we analyzed the chickens’ posture based on the above two types of images. The colored image was used for image correction, and the depth image was used to locate the key area under the feeding trough and identify the posture of the chickens. This method proves to be effective in detecting the chickens’ posture even under poor light conditions.

The present research aims to develop this methodology to detect caged chickens and identify both their standing and lying posture. The main objectives of this work are as follows:

1. To accurately find the feeding trough position, this paper proposed an image correction method to rotate the image to make the trough in the image horizontal, so as to solve the problem that the position of the trough could be accurately obtained due to the roll angle when the area was photographed.
2. To accurately identify the position of the trough, the variance method and the speeded-up robust features method were proposed to identify the trough according to its features, and the key area was obtained indirectly through the position of the trough.
3. To accurately identify the posture of the chickens, 3D information from the depth camera was used to extract the chickens from the key area in the image. The chickens were detected by some constraint conditions, and finally, the standing and lying positions of the chickens were automatically recognized.

## 2. Materials and Methods

### 2.1. Experimental Environment

**Figure 1:**
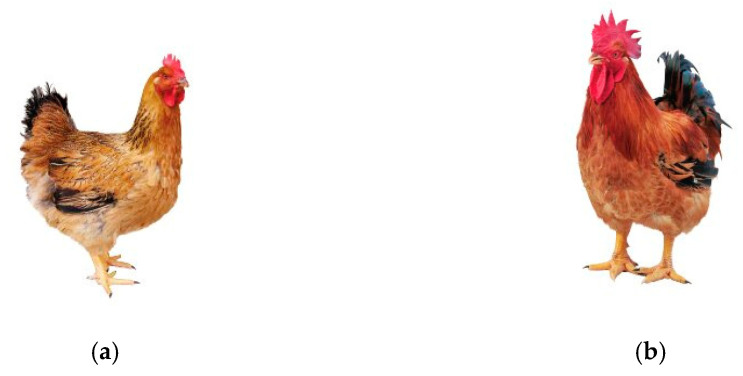
(**a**) Female and (**b**) male K90 jute broilers..

We have studied a breeding flock of K90 jute broilers (Figure 1) and White Recessive Rock (WRRC) white broilers at the production base in Foshan, China; the farm has 400,000 broilers. Among them, K90 was cultivated by cross-breeding technology. The paternal line is the Guangdong jute chicken, which is native to China, and the maternal line is the Kabir recessive broiler, which is native to Israel [24].

**Figure 2:**
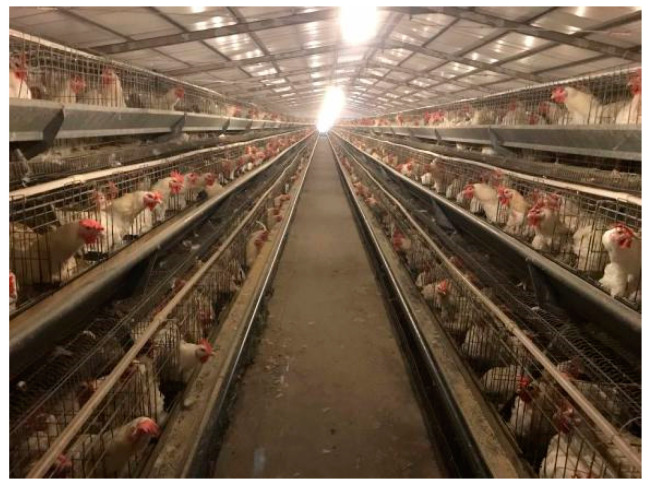
The production base in Gaoming District, Foshan City, Guangdong Province.

In our experiment, the K90 chickens were between 40 and 70 weeks old with a mass between 2.8 and 3.8 kg. The WRRC were between 40 and 70 weeks old with a mass between 3.2 and 4.2 kg. These were breeding birds, and were not being prepared for consumption, as shown in Figure 2. This experiment was performed in accordance with the guidelines approved by the Experimental Animal Administration and ethics committee of South China Agriculture University (SYXK-2019-0136).

Each cage in the farm is limited to two broilers to ensure that the broiler receives a certain amount of feed per meal and controls its weight. In order to improve the generalization ability of the algorithm, we randomly photographed broilers from 09:00 to 17:00. The lighting in the farm mainly relies on artificial lighting, though some chicken cages near the fan are affected by daylight. The lux level and color of the lighting were nearly 40 ± 7 lx and yellow. Due to the complex environment of the farm, it is difficult to effectively separate the broilers from the background with a traditional monocular camera. Therefore, an Asus Xtion Pro Live depth camera (ASUS TeK Computer Inc., Taipei, Taiwan) was used in this paper. The camera has a horizontal viewing angle of 58°and a vertical viewing angle of 45°. It can simultaneously acquire color and depth images with a resolution of 640 × 480, and the frame rate is 30 FPS.

### 2.2. Data Processing

**Figure 3:**
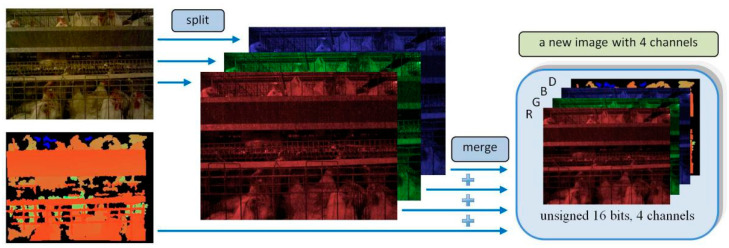
Image acquisition. The color image is divided into three single-channel images of the R component, the G component, and the B component, and the three components are merged with the deep image into a four-channel image for storage.

Since the depth camera simultaneously outputs the color image and the depth image, for the convenience of saving the acquired image material, the two types of images were merged, and finally, a file in portable network graphics (PNG) format was generated, as shown in Figure 3.

The color image acquired by the depth camera was 8-bit 3-channel, and the acquired depth image was 16-bit single channel. In order to ensure that the merged image did not lose the original data, the color image was converted into 16-bit 3-channel, and then merged with the depth image; finally, a 16-bit 4-channel PNG format image was generated. At this moment, the generated image contained both color image and depth image information. The Xtion depth camera was placed on the carrying device, and the carrying device moved at a constant speed of 0.1 m/s. In this paper, 2504 images were randomly selected from the captured video to manually mark the posture of the broilers and prepare for the performance test of the subsequent algorithm.

### 2.3. Algorithm Framework and Implementation Steps

**Figure 4:**
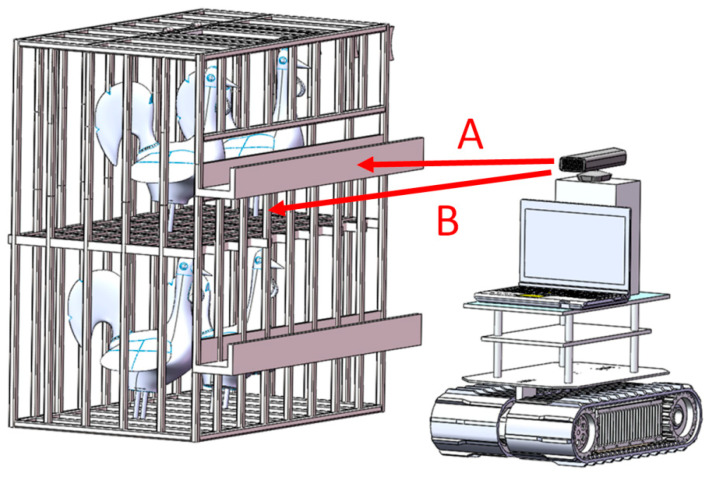
Arrow A stands for the feeding trough recognition and distance calculation between the camera and the feeding trough; Arrow B represents the key area location and the prostrate broiler recognition within this area.

Since the feeding trough blocked most of the body of the broiler, when the broiler was standing, only the broiler’s foot could be seen under the feeding trough. When the broiler was lying down, the profile of the broiler could be seen under the feeding trough. Based on this difference, a set of algorithms was designed for detecting the posture of the broilers. The depth camera was mounted on the carrying device for all-day running, as shown in Figure 4.

Thus, the problem to be solved in this paper was how to analyze whether the broiler was in a lying posture in the captured image.

**Figure 5:**
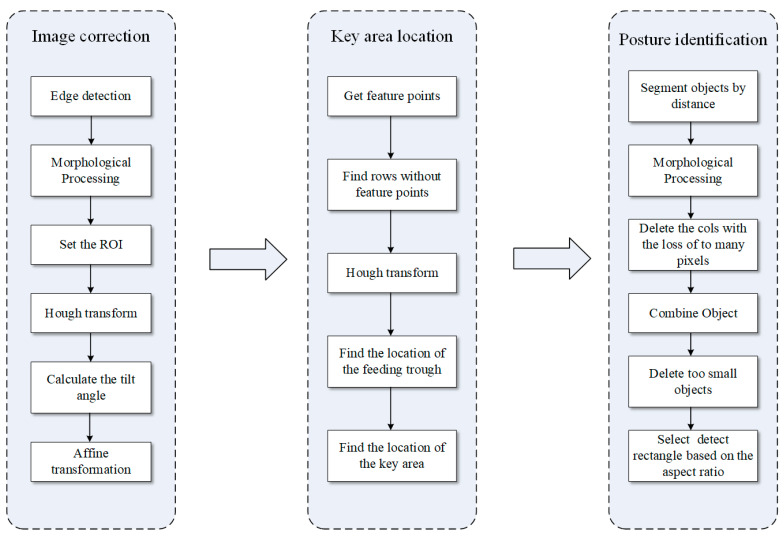
Algorithm flow chart.

The algorithm was mainly divided into three parts: image correction, key area location, and posture identification. The algorithm flow chart is shown in Figure 5. Image correction was used to solve the problem that the camera rotated due to the roll angle caused by the camera shaking. After the correction, the feeding trough was in a horizontal state, ready for subsequent image segmentation. The key area location was used to find the exact position of the feeding trough and then delineate the area right beneath the feeding trough, where the camera could capture a picture of the broiler’s lying posture. This key area was used to split the broiler from the background. The posture identification was used to identify the prostrate broilers by using the depth information in the key area.

#### 2.3.1. Image Correction

**Figure 6:**
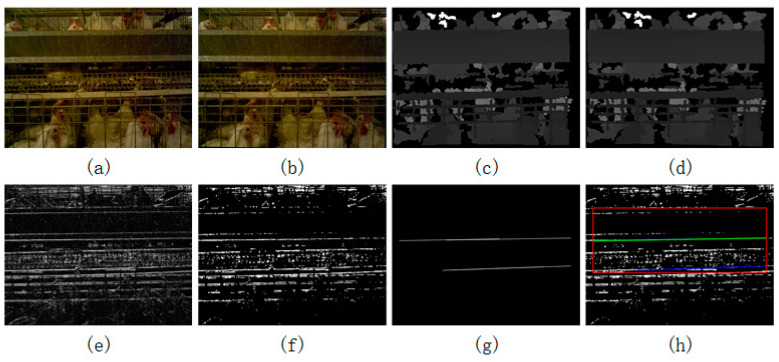
Rotation of the original image for correction. (**a**,**c**) The color and depth images before correction; (**b**,**d**) the color and depth images after correction; (**e**) the effect of under edge detection; (**f**) the effect of morphological processing; (**g**) the effect of the first Hough transform; and (**h**) the final effect.

The accurate extraction of the position of the trough was the premise and guarantee for the positioning of the prostrate broiler. To this end, this section is dedicated to rotating the image so that the trough could be in a horizontal position by the image correction method. This step could simplify subsequent algorithms. Since the camera was mounted on the carrying device, the shaking of the carrying device prevented the camera from capturing the trough in a horizontal state. The color image and depth image captured are shown in Figure 6a,c. It can be seen that the two images are tilted slightly counterclockwise, so this paper proposes an image correction method for the calibration of the images of the caged broilers. The specific steps are as follows.

(a1) Change the colored image in Figure 6a into a grayscale image, and the edge detection is performed in the y-direction by using a 3 × 3 Sobel operator to obtain a series of straight lines, as shown in Figure 6e;
(a2) Figure 6e is transformed with a median filter of scale 3, the noise points in the image are removed, and a binarization operation with a threshold of 100 is performed, as shown in Figure 6f;
(a3) Set a region of interest (ROI). The point in the upper left corner of the rectangle is (x = 25, y = 80), and the point in the lower right corner is (x = 615, y = 300);
(a4) The Hough transform is used to extract the straight-line segment of the ROI in Figure 6f and is drawn in a full black image to obtain Figure 6g. At this time, there are still several straight-line segments in the figure;
(a5) For Figure 6g, the Hough transform is used again to extract the straight-line segment, which is represented by the blue line, and the longest straight-line segment is indicated by the green line, as shown in Figure 6h;
(a6) Determine if the green line is found; if not, skip to step a9; if found, calculate the tilt angle of the green line. The green line in Figure 6h is inclined counterclockwise by 0.978°;
(a7) Calculate the rotation matrix for the tilt angle obtained in step a6, wherein the rotation matrix in Figure 6h is $\left[\begin{array}{c} 1.000,\;-0.017,\;4.144\\ 0.017,\;1.000,\;-5.428 \end{array}\right]$;
(a8) Use the affine transformed in Figure 6a,c according to the rotation matrix in step a7 to achieve image correction. The corrected results are shown in Figure 6b,d;
(a9) End the image correction algorithm.

The algorithm uses the features of the trough edge to find the tilt angle of the image to calibrate the original image. Subsequent algorithms need to locate the area beneath the trough to find the broiler, so it is especially important to adjust the trough in the image to a horizontal state. The position of the trough in the image does not change too much, and the ROI can be preset to reduce the amount of calculation, as shown by the red rectangle in Figure 6h. Since the edge of the trough is distinct and linear, the angle of inclination of the trough can be obtained by a Hough linear transformation after edge extraction in the y-direction. The Hough transform needs to count the number of pixels in the image, and the algorithm first performs median filtering to eliminate noise. To ensure that the trough is as horizontal as possible after the rotation, the algorithm selects the straight line in the ROI to calculate the inclination angle. The algorithm uses Hough transforms two times to obtain the final line, because the first time using Hough may cause the same line to be divided into several segments, resulting in the failure of the algorithm to find the longest line. Therefore, the algorithm performs the Hough transform for the first time to screen for longer straight lines, and it draws all the selected lines in an all-black image, and it then uses the Hough transform again in this image to obtain the longest straight line. The straight line can properly describe the inclination of the trough, and the affine transformation, according to the inclination angle, enables the trough to stay horizontal in the image.

#### 2.3.2. Key Area Location

**Figure 7:**
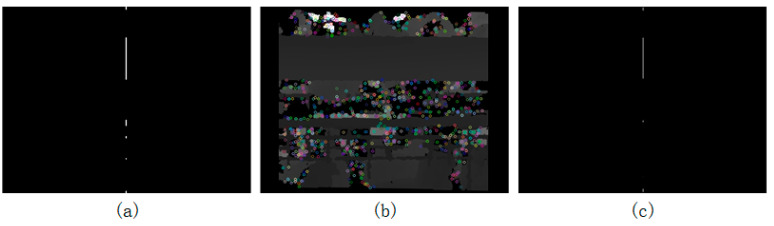
Trough location. (**a**) The variance method to find the trough area; (**b**,**c**) The SURF method to find the trough area.

In this paper, the specific area beneath the trough was used as the key area. To identify the key area, the trough must first be accurately identified. In the color image, the pattern in which the color of the trough was affected by the light was not clear, and there were various noises. Thus, it was difficult to accurately identify the trough from the color image. Since the plane of the trough was relatively flat, the distance from the neighboring points on the trough to the camera did not change much. Therefore, the color displayed by the trough in the depth image was relatively simple, as shown in Figure 7b. According to this feature, two methods for extracting the trough using the depth image were proposed. As the depth image and the color image were registered, the left and right sides of the depth image each had a black border with fixed width. In order to prevent the black edge from interfering with the trough extraction, the algorithm only took image data between the 50th column from the left to the 50th column from the right.

Use the variance method to find the trough area:

(b1) Calculate the variance of the pixel value of each row in the depth image, and mark the row if the variance of the row is less than a certain threshold;
(b2) Create a new all-black image of the same size as the depth image, and set the midpoint of the marked line to white, as shown in Figure 7a;
(b3) Use the Hough transform to find all the straight-line segments in step b2;
(b4) Find the longest straight-line segment in step b3. The area constituted by the collection of rows that each point on the straight-line segment represents is the trough region.

**Figure 8:**
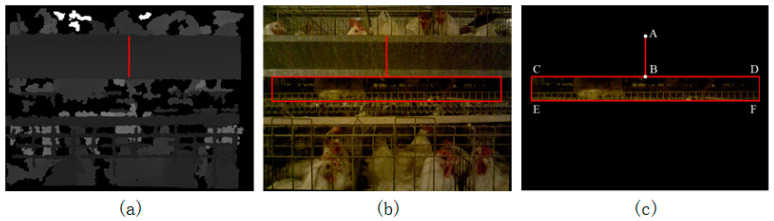
Key area location. (**a**) The area where the trough is located is shown with a red vertical line segment in the depth image; (**b**) the area where the trough is located is shown with a red vertical line segment in the color image, and the key area is marked with a red rectangle; (**c**) the image of the key area after extraction.

Use the SURF method to find the trough area:

(c1) Define the Hessian threshold in SURF. The default threshold is 400;
(c2) Obtain the SURF feature points in the depth image, as shown by the colored hollow points in Figure 7b;
(c3) Mark both the line in which the feature point lies and its neighboring lines (upper and lower lines);
(c4) Create a new all-black image of the same size as the depth image, and set the midpoint of the unmarked line to white, as shown in Figure 7c;
(c5) Use the Hough transform to find all the straight-line segments in step c4;
(c6) Find the longest straight-line segment in step c5. The area constituted by the collection of rows that each point on the straight-line segment represents is the trough region, as shown by the red vertical line in Figure 8a.

As can be seen from Figure 8b, if the broiler was in a lying posture, most of the body contours could be seen below the trough. Therefore, the algorithm searched for the key area (the red rectangle CDEF in Figure 8c) starting from the lowermost end of the red vertical line segment representing the trough (point B in Figure 8c). Due to the influence of errors during the forward movement of the carrying device, the distance between the camera and the cage could not be kept constant, so there was a difference in the size of the trough between the captured images. The height of the key area and the trough satisfied the following relationship in this paper:

(1) $$l_{CE}=l_{DF}=k_{1}l_{AB}$$

(2) $$l_{CD}=l_{EF}=590$$

where l represents the length of the line segment between two points, measured in pixels, and $k_{1}$ is a fixed parameter. The resolution of the image used in this paper was 640 × 480. Since there were unrecognizable black edges on the left and right sides of the depth image, 25 pixels on each side were left unrecognized, so the length of the red rectangle was 590 pixels.

The closer the distance between the camera and the caged broiler, the longer the red vertical line segment representing the trough and the larger the key area. The farther the distance between the camera and the caged broiler, the shorter the red vertical line segment representing the trough and the smaller the key area. Therefore, the above equation can ensure that the algorithm dynamically adjusts the height of the key area as the size of the trough changes, thereby improving the stability of the algorithm.

#### 2.3.3. Posture Identification

The key issue to be solved in this paper was how to find the lying posture of the broilers. The key area was blocked by the feeding trough, and the light was insufficient. It was difficult to accurately and efficiently identify the lying broilers in a colored image without using supplementary light. For this purpose, the depth image was used to identify the lying broiler in the image. The first two sections completed the necessary preliminary work: (1) the image was calibrated so that the trough would be horizontal in the image; and (2) the trough was accurately identified and the key area was found. Based on these two points, this section proposes a method to accurately and efficiently identify the lying posture of the broilers.

**Figure 9:**
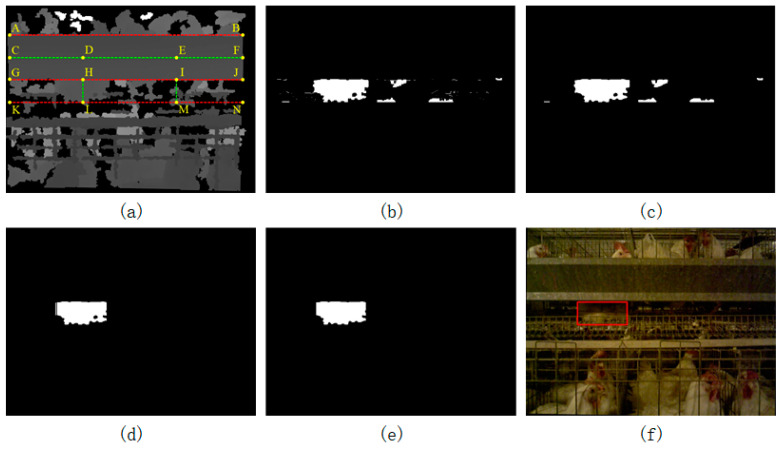
Posture identification algorithm steps. (**a**) The process of extracting the object from the depth image through distance information, wherein the rectangle ABGJ was the identified trough and the rectangle GJKN was the key area. For example, after the real distance from point D to the camera was obtained in (**a**), the true distance from all points on HL to the camera were obtained to compare with the real distance of point D. If it satisfied Equation (3), it was considered that the point on the line segment HL was the foreground point. Otherwise, it was the background point. Similarly, after obtaining the true distance from point E to the camera, all the points on the line segment IM were judged to segment the image. (**b**) The result of segmentation of the image; (**c**) the result of the morphology process; (**d**) the result after deleting columns with fewer white pixels; (**e**) the result of combining multiple contours with close distances; and (**f**) the final result of identifying the broiler in a lying posture.

Figure 9a is a corrected depth image. The rectangle ABGJ represents the identified feeding trough, and the rectangle GJKN represents the key area. When the broilers were standing, only legs could be seen in the key area beneath the trough; thus, only a small area of obstruction would be produced in the key area. If the broilers were lying down, their body contours would generate a large area of obstruction in the key area. Therefore, as long as a large area of obstruction was found, it could be judged that the broiler was in a lying posture. The depth image could segment the foreground and background according to the distance. Since the camera was moving at every moment, the distance between the camera and the broiler was not constant, which made the broiler division more difficult. Therefore, this paper used the distance from the camera to the trough to estimate the distance between the camera and the broiler in the key area to achieve dynamic threshold segmentation.

The segmentation algorithm is as follows:

(d1) The trough has been corrected into a horizontal state. Then, find the horizontal line in the middle of the trough, as indicated by the green line segment CF in Figure 9a;
(d2) Use the depth image to obtain the true distance information $d_{c}$ of every point on the green line segment CF from left to right;
(d3) Find all the points in the same column as the points on CF in step d2 from the key area (rectangle GJKN), and judge whether it satisfies Equation (3);
(d4) Set the points that satisfy Equation (3) in the key area to the foreground (white) and the points that are not to the background (black). The result is as shown in Figure 9b;
(d5) Filter out the noise points by the morphological method (opening and closing operation and median filtering), and the result is shown in Figure 9c;
(d6) Use Equation (4) to estimate each column in the image. If the equation is satisfied, keep the column pixel distribution as it is. Otherwise, set all the pixels in the column to black. As shown in Figure 9d, this step can wipe out columns with fewer white pixel points (suspected targets);
(d7) Combine contours with distances less than 20 pixels to prevent the same target from being divided into multiple result boxes. The result is shown in Figure 9e;
(d8) Find the contours of all the white areas in the image, delete the contours that are too small, and find the circumscribed rectangle of the remaining contours;
(d9) If the circumscribed rectangle satisfies Equation (5), retain the rectangle; otherwise, delete it. The rectangles meeting the condition are the final area where the lying broiler is located, and the result is shown in Figure 9f.

Equation (3) is used to locate the area to be detected.

(3) $$d_{c}+k_{2}<d_{c}^{\prime }<d_{c}+k_{3}$$

where $d_{c}$ is the real distance between the point in the column c on line CF and the camera; $d_{c}^{\prime }$ is the true distance between the point in the column c on rectangle GJKN and the camera; and $k_{2}$ and $k_{3}$ are fixed parameters.

Equation (4) is used to delete columns with fewer white pixels.

(4) $$N_{c}>\frac{H_{roi}}{k_{4}}$$

where $N_{c}$ is the number of white pixel points in the column c; $H_{roi}$ is the height of the key area (rectangle GJKN); and $k_{4}$ is a fixed parameter.

Equation (5) identifies the area where the lying broiler.

(5) $$\frac{W_{rect}}{H_{rect}}>k_{5}$$

where $W_{rect}$ is the width of the circumscribed rectangle; $H_{rect}$ is the height of the circumscribed rectangle; and $k_{5}$ is a fixed parameter.

For step d3, the reason for satisfying Equation (3) is that the distance to the cage from the camera was slightly larger than the distance to the trough from the camera. At the same time, because there may have been a pitch angle between the camera and the cage, setting a threshold range could better extract the target of the lying broiler. The reason for using the midpoint on each column of the trough to divide the pixels in the same column of the key area was that the distance from the midpoint to the camera on the different columns on the trough was different. If the camera was facing right in front of the broiler cage, the trough in the center of the image was closer to the camera, and the trough at the edge of the image was farther from the camera. The situation was more complicated when the camera and the cage had a yaw angle. Therefore, the segmentation of each column in the key area was controlled by the midpoint of the same column of the trough to avoid the segmentation error caused by the distance factor, and the dynamic threshold segmentation was realized.

**Figure 10:**
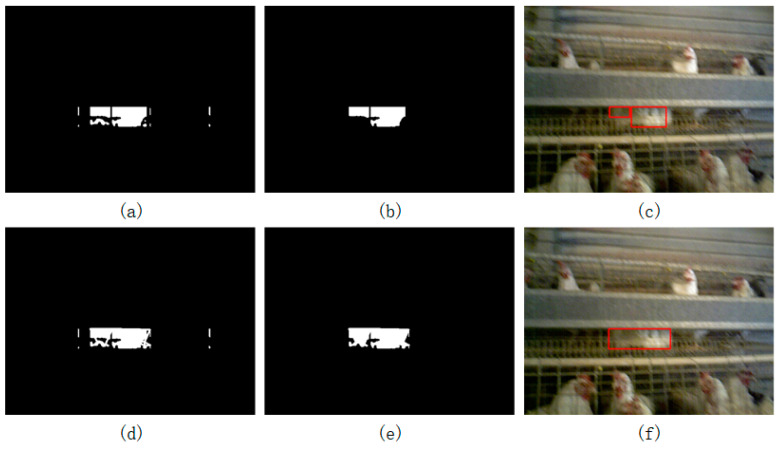
Combining objects. (**a**) The result after performing step d6; (**b**) the result of directly performing step d8, skipping step d7 based on (**a**); (**c**) the result of performing step d9 based on (**b**); (**d**) the result after performing step d7 based on (**a**); (**e**) the result after performing step d8 based on (**d**); and (**f**) the result after performing step d9 based on (**e**).

Due to the complexity of the environment, the same object could be divided into several blocks during the object extraction process, as shown in Figure 10a. The function of step d7 was to recombine these separated parts. The algorithm used the distance relationship between all the suspected objects to determine whether the objects belonged to the same object. If the distance between them was less than a certain threshold (set to 20 pixels in this paper), they were considered to be the same object, and the two objects were connected together by a thin line, as shown in Figure 10d. It should be noted that the algorithm determines the distance between the objects by determining the shortest distance between the points on the edge contours of the two objects and cannot directly use the distance between the centers of gravity of the two objects. This is because when the two objects are large, the algorithm will conclude that the two objects are far apart when using the centers of gravity, describing the distance as the radius of the circumcircle of the two objects as large, and these two objects would not be connected. However, these two objects may probably be almost glued together and should be determined to be the same object. Figure 10c shows the result without step d7, and Figure 10f shows the result using step d7. As can be seen from the figure, the broiler object is divided into left and right halves without step d7, and after adding step d7, the broiler can be more accurately identified.

**Figure 11:**
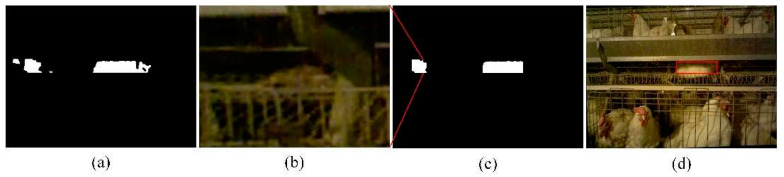
Finding a satisfactory rectangular object. (**a**) The result after performing step d5; (**b**) the design of the pillar; (**c**) the result after performing step d8; and (**d**) the result after performing step d9.

The posture identification algorithm could identify the lying broilers in the key area. However, it would also misidentify the pillars between cages, as shown in Figure 11c. After step d8, the pillars between the broiler cages were also misidentified. Step d9 used the relationship between the height and width of the rectangle to remove the interference factors of the pillars. The lying broiler was flat in the key area, while the pillars were elongated. The interference could be filtered out by removing the elongated target. As shown in Figure 11d, after performing step d9, the pillar was removed.

The posture identification algorithm used the distance between the trough and the camera to estimate the position of the lying broiler in the key area. After the target was extracted from the background, the morphological method was used to remove the noise point, and then step d6 was used to delete columns with fewer white pixels, thereby removing some interference caused by the edge of the key area. Step d7 was used to optimize the target area by merging the areas of the same target, and step d8 was used to delete the targets with too small of an area after optimization. In step d9, the interference factors of the pillars were removed, and the final results were achieved. It can be seen that after a series of algorithm optimizations, most of the interference factors were removed, which could improve the recognition accuracy.

### 2.4. Evaluation Index

This study evaluated the results of the proposed algorithm using accuracy, precision, recall, and *F*1 scores, as shown in Equations (6)–(9):

(6) $$A=\frac{TP+TN}{TP+TN+FP+FN}$$

(7) $$P=\frac{TP}{TP+FP}$$

(8) $$R=\frac{TP}{TP+FN}$$

(9) $$F1=\frac{2\cdot P\cdot R}{P+R}=\frac{2TP}{2TP+FP+FN}$$

where *A* is accuracy; *P* is precision; *R* is recall; *F*1 is the *F*1 Score; *TP* is the true-positive rate; *TN* is the true-negative rate; *FP* is the false-positive rate; and *FN* is the false-negative rate.

## 3. Results and Discussion

### 3.1. Selection of Parameters and Methods

**Figure 12:**
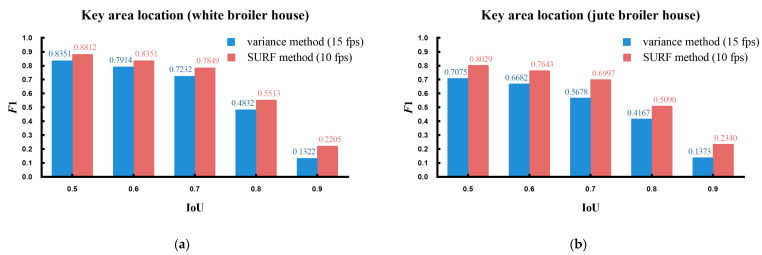
Comparison of the key area location algorithm. *F*1 means *F*1 score. (**a**) An algorithm test for the white broiler house and (**b**) an algorithm test for the jute broiler house.

In the Materials and Methods section, a key area location algorithm was introduced which included using the variance method and the SURF method to find the feeding trough. In this section, the *F*1 score was used to measure the recognition effect of the algorithm, as shown in Equation (9). The *F*1 score is an indicator of the precision ratio and recall ratio. The higher the *F*1 score, the better the recognition of the algorithm. Figure 12 is a comparison of the two methods. It can be seen that the *F*1 score of the SURF method was usually larger than that of the variance method under different IoUs, whether in the white broiler house or in the jute broiler house. Therefore, the SURF method was better than the variance method. The processing speed could achieve 15 fps using the variance method and ten fps with the SURF method when dealing with images with a resolution of 640 × 480 on an i5-8500 CPU with 3.20 GHz per core. Therefore, in terms of speed, the variance method was better. However, since the speed of 10 fps already met the requirements for posture identification, the SURF method was chosen for finding the feeding trough.

**Figure 13:**
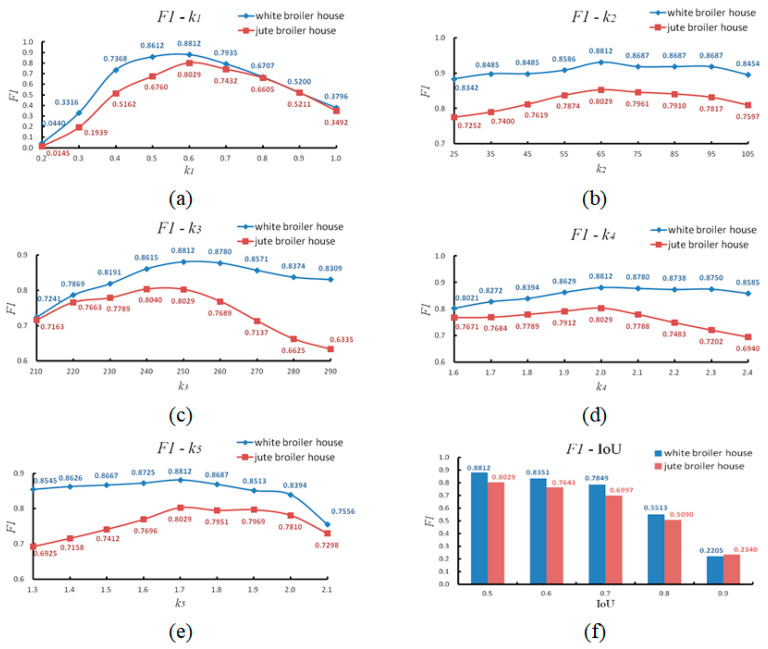
The influence of the parameters k1, k2, k3, k4, and k5 on the detection effect of the algorithm. Blue indicates the detection effect of the white broiler house, and red indicates the detection effect of the jute broiler house. (**a**–**e**) The correlation between one of the parameters and the *F*1 score while keeping the other four parameters optimal (k1 = 0.6, k2 = 65, k3 = 250, k4 = 2.0, k5 = 1.7); (**f**) the *F*1 score at different IoUs when all five parameters were optimal.

Five fixed parameters were mentioned in the Section 2, namely k1, k2, k3, k4, and k5. The parameter k1 was used to adjust the height of the key area beneath the trough, the parameter k2 and the parameter k3 were used to adjust the segmentation range of broilers, the parameter k4 was used to adjust the filter range, and the parameter k5 was used to adjust the threshold of the aspect ratio of the final object. The selection of these five parameter values affected the recognition effect of the algorithm, so it was necessary to find the optimal solution of these five parameters. Since these five parameters have practical meanings, the initial value could be artificially preset. In addition, the parameter values could be adjusted according to the recognition effect. A computer was then used to enumerate all the combinations of the five parameters near the initial value. The F1 score was used as the recognition criterion to obtain the optimal values for these five parameters. It could be found that when k1 = 0.6, k2 = 65, k3 = 250, k4 = 2.0, and k5 = 1.7, the highest F1 score was obtained. Figure 13 demonstrates the correlation between one of the parameters and the F1 score while keeping the other four parameters unchanged by the control variable method.

As can be seen from Figure 13a, when k1 was 0.6, the white broiler house and the jute broiler house had the highest F1 score. As can be seen from Figure 13b, when k2 was 65, the white broiler house and the jute broiler house had the highest F1 score. As can be seen from Figure 13c, when k3 was 250, the white broiler house had the highest F1 score. When k3 was 240, the jute broiler house had the highest F1 score (Since the k3 in the jute broiler house was 240 or 250, the difference was small. In order to consider the case of the white broiler house, k3 was 250 here). As can be seen from Figure 13d, when k4 was 2.0, the white broiler house and the jute broiler house had the highest F1 score. As can be seen from Figure 13e, when k5 was 1.7, the white broiler house and the jute broiler house had the highest F1 score. Therefore, the above values were taken as the optimal values of the five parameters. Figure 13f shows the F1 scores for the algorithm under different IoUs. It can be seen that when IoU > 0.8, the decrease of the recognition effect of the algorithm was more obvious. Table 1 shows the evaluation of the algorithm under different IoUs.

In general, the recognition effect of the algorithm for a white broiler was better than for a jute broiler (yellow and black feathers). This is because the white broiler in the house is bulky, while the jute broiler is relatively small. For larger white broilers, only their legs can be seen when standing, and most of the body contours can be seen when the broilers are lying down. In some small-sized jute broilers, the broiler legs are relatively short, so their body parts can still be detected in the key area when they are standing, resulting in identification errors.

### 3.2. Algorithm Stability Test

**Figure 14:**
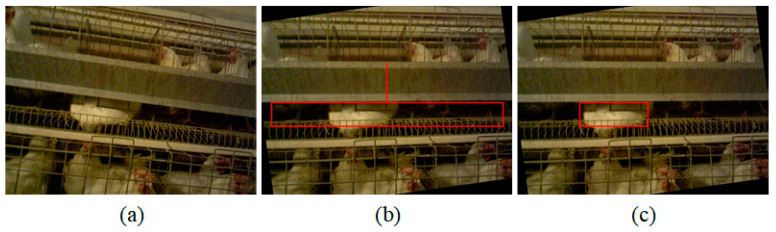
Algorithm stability test at large tilt angles. (**a**) The original color image; (**b**) the image after correction; and (**c**) the effect of image correction on the lying broiler identification.

In order to test the stability of the algorithm, some images with poor quality were chosen for verification. Figure 14 shows the images in the case where the camera had a large roll angle. As shown in Figure 14a, as the trough was severely tilted, it was not possible to accurately locate the lying broiler without image correction. Figure 14b shows a rotation correction of the image using the image correction method described above. As can be seen from the figure, the trough has been corrected to a horizontal state. Figure 14c is the effect on the identification of lying broilers. After the image correction, the posture identification algorithm described in this paper can be used to detect the lying broiler well.

**Figure 15:**
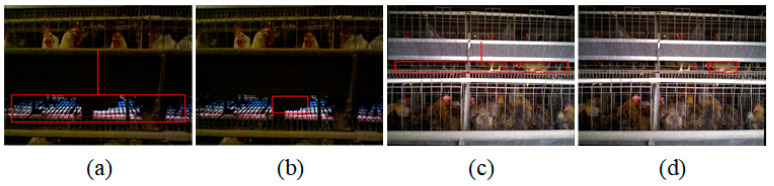
Algorithm stability test under different ambient light. (**a**,**b**) Cages that were not exposed to sunlight; (**c**,**d**) cages that were exposed to direct sunlight near the window; (**a**,**c**) the effect on the key area location; and (**b**,**d**) the effect on the lying broiler identification.

In order to test the influence of natural light on the algorithm, the cage lacking light (less than 10 lux) and direct sunlight (above 40 lux) was selected to test the stability of the algorithm, as shown in Figure 15. As can be seen from Figure 15a,b, even if the image was dim, the lying white broiler could be accurately identified. This was because the algorithm used a combination of the color image and depth image. In particular, the algorithm for identifying broilers was done using a depth image. Therefore, light was not necessarily required, and the algorithm could adapt well to the environment of the broiler house. Figure 15c,d shows the recognition effect in the case where there was strong direct sunlight in a part of the image. The right side of the image is brighter, and the left side is darker, but the jute broiler lying down in the image could be accurately identified.

(10) $$\mathrm{Status}=\left\{\begin{array}{c} \frac{photographed\;time}{detection\;frequency\times time}<50\%\;,\;normal\\ \frac{photographed\;time}{detection\;frequency\times time}\geq 50\%\;,\;abnormal\;or\;atrophy \end{array}\right.$$

However, caged chickens slept for about eight hours, after the lights were turned off, except for one chicken that was too old and needed more rest. Other circumstances require attention or, at least, they could appear to be sleeping too much. As shown in Equation (10), if the same broiler spent more than 50% of its photographed time in a lying posture within one daytime, the broiler would be considered to be in a state of atrophy or abnormal. Finally, an alarm would be raised to prompt the staff to further examine the location of the chicken by the current positioning of the inspection robot and the QR code and other marks on the chicken coop.

In this study, we proposed an algorithm for the automatic detection of lying and standing chickens. The algorithm could well recognize the posture of the chicken in the image; however, the algorithm could not tell whether the birds were resting or sick. If the chicken is completely covered by the trough, our method cannot solve this problem. We can combine other information such as sound [25] and temperature [26], which will also make it possible to determine whether the chickens are sick or not.

Meanwhile, the increased mortality and culling rates, from an animal welfare perspective, suggest that the conditions in caged systems may not meet the chickens’ basic physiological and psychological needs. Poor housing conditions, such as inadequate ventilation, overcrowding, and suboptimal nutrition, often exacerbate health issues and lead to welfare compromises. This also raises ethical concerns, as higher mortality rates may reflect substandard care, and pushing for stronger regulations or even a shift toward cage-free systems would ensure better welfare outcomes [27].

In our future work, we will design different types of broiler cages to make the algorithm of caged broilers analyze the system more generally. At the same time, the algorithm will be improved to adapt to different types of broilers and to extend the scope of recognition to broiler feeding [28], drinking [29], and other behaviors [30,31,32].

## 4. Conclusions

This paper proposed a posture detection method for caged broilers based on computer vision. This method identified the position of the trough and obtained the key area after using image correction, and then depth image was used to extract the broilers from the background in the key area and finally identified the broilers in a lying posture. The results show that the algorithm can achieve 97.80% precision and 80.18% recall (IoU > 0.5) in the white broiler house and can achieve 79.52% precision and 81.07% recall in the jute broiler house (IoU > 0.5). The algorithm runs at ten fps on an i5-8500 CPU (resolution 640 × 480 image), which can meet the requirements of lying broiler detection. In conclusion, the work described in this paper solved the problem of insufficient light (less than 10 lux) without changing the environment and identified the standing and lying posture of the broiler in a complex cage environment. In addition, developing this method will help farmers monitor their animals’ behavior and health more efficiently.

## Figures and Tables

**Table 1:** The evaluation of the algorithm under different IoUs.

IoU	Accuracy (*W*)	Precision (*W*)	Recall (*W*)	Accuracy (*Y*)	Precision (*Y*)	Recall (*Y*)
0.5	0.8367	0.9780	0.8018	0.7778	0.7952	0.8107
0.6	0.7823	0.9759	0.7297	0.7425	0.7817	0.7476
0.7	0.7279	0.9733	0.6577	0.6883	0.7571	0.6505
0.8	0.5238	0.9556	0.3874	0.5556	0.6641	0.4126
0.9	0.3265	0.8750	0.1261	0.4146	0.4342	0.1602

*W* represents the recognition in the white broiler house; *Y* represents the recognition in the jute broiler house.

## Data Availability

The data presented in this study are available on request from the corresponding author.
